# 

**Published:** 1904-06

**Authors:** 


### BOOKS RECEIVED.

Manual of Materia Medica and Pharmacy. Specially designed for the use of Practitioners and Medical, Pharmaceutical, Dental, and Veterinary Students. By E. Stanton Muir, Ph.G., V.M.D., Instructor in comparative Materia Medica and Pharmacy in the University of Pennsylvania. Third edition, revised and enlarged. Octavo, 192 pages. Philadelphia: F. A. Davis Company. 1904. (Price, $2.00 net.)

Lea's Series of Medical Epitomes. Pediatrics. A Manual for Students and Practitioners. By Henry Enos Tuley, A.B., M.D., Professor of Obstetrics in the Medical Department of Kentucky University, Louisville. Duodecimo, pp. 266, with 33 engravings. Series edited by V. C. Pedersen, M.D., Instructor in Surgery at the New York Polyclinic Medical School and Hospital. New York, Philadelphia: Lea Brothers & Co. 1903. (Price, $1.00.)

The Medical News Pocket Formulary. By E. Quin Thornton, M.D., Assistant Professor of Materia Medica in the Jefferson Medical College, Philadelphia. New (sixth) edition. Leather, wallet shape for the pocket. Philadelphia and New York: Lea Brothers & Co. 1904. (Price, $1.50 net.)

A System of Practical Surgery. By Prof. E. von Bergmann, M.D., Berlin; Prof. P. von Bruns, M.D., Tubingen, and Prof. J. von Mikulicz, M.D., Breslau. Volume II. Surgery of the Neck, Thorax and Spinal Column. Translated and edited by William T. Bull, M.D., Professor of Surgery, College of Physicians and Surgeons, New York, and Carlton P. Flint, M.D., Instructor in Minor Surgery. Royal octavo, pp. 820. Illustrated. Lea Brothers & Co., New York and Philadelphia. 1904. (Price: cloth, $6.00; leather, $7.00; half morocco, $8.50 net.)

A Practical Treatise on Medical Diagnosis for Students and Practitioners. By John H. Musser, M.D., Professor of Clinical Medicine in the University of Pennsylvania. Fifth edition, revised and enlarged. Octavo, pp. 1213; 395 engravings and 63 colored plates. Philadelphia and New York: Lea Bros. & Co. 1904. (Price: cloth, $6.50; leather, $7.50; half morocco, $8.00 net.)

International Clinics. A Quarterly of Illustrated Clinical Lectures and especially prepared original articles on Treatment, Medicine, Surgery, Neurology, Pediatrics, Obstetrics, Gynecology, Orthopedics, Pathology, Dermatology, Ophthalmology, Otology, Rhinology, Laryngology, Hygiene and other topics of interest to students and practitioners. By leading members of the medical profession throughout the world. Edited by A. O. J. Kelly, A.M., M.D., Philadelphia. Volume I. Fourteenth series. 1904. Philadelphia: J. B. Lippincott Company. (Cloth, $2.00.)

Röntgen Ray Diagnosis and Therapy. By Carl Beck, M.D., Professor of Surgery in the New York Post-Graduate Medical School and Hospital; Visiting Surgeon to Saint Mark's Hospital and the German Poliklinik. Octavo, pp. 479, with 322 illustrations. New York and London: D. Appleton & Co. 1904. (Price, $4.00.)

A Guide to the Clinical Examination of the Blood for Diagnostic Purposes. By Richard C. Cabot, M.D. Octavo, pp. 569. Illustrated. Fifth revised edition. New York: William Wood & Co. 1904. (Price, $3.50.)

Graves's Disease with and without Exophthalmic Goitre. By William Hanna Thomson, M.D., LL.D., Physician to the Roosevelt Hospital, New York. Octavo, pp. 143. New York: William Wood & Co. 1904. (Price, $1.50.)

Proceedings of the American Medico-Psychological Association at the fifty-ninth annual meeting held at Washington, D. C., May 12-15, 1903. C. B. Burr, M.D., Secretary. Published by the Association. 1903.

A Manual of Fever Nursing. By Reynold Webb Wilcox, M.D., Professor of Medicine in the New York Post-Graduate Medical School and Hospital. Duodecimo, pp. 236. Philadelphia: P. Blakiston's Son & Co. 1904.

The Practical Medicine Series of Year Books. Ten volumes. Issued under the general editorial charge of Gustavus P. Head, M.D., Professor of Laryngology and Rhinology in the Chicago Post-Graduate Medical

School. Volume IV. Gynecology. Edited by E. C. Dudley and William Healy, Chicago. Duodecimo, pp. 216. Chicago: The Year Book Publishers. 1904. (Price, $1.50; entire series, $5.50, payable in advance.)

